# Inhibition of Colony-Stimulating Factor 1 Receptor by PLX3397 Prevents Amyloid Beta Pathology and Rescues Dopaminergic Signaling in Aging 5xFAD Mice

**DOI:** 10.3390/ijms21155553

**Published:** 2020-08-03

**Authors:** Yeonghoon Son, Ye Ji Jeong, Na-Rae Shin, Se Jong Oh, Kyung Rok Nam, Hyung-Do Choi, Jae Yong Choi, Hae-June Lee

**Affiliations:** 1 Division of Radiation Bioscience, Korea Institute of Radiological & Medical Sciences, Seoul 01812, Korea; sonyh@kribb.re.kr (Y.S.); whyj0914@kirams.re.kr (Y.J.J.); tlsskfo870220@gmail.com (N.-R.S.); 2 Primate Resources Center, Korea Research Institute of Bioscience and Biotechnology (KRIBB), Jeonbuk 56216, Korea; 3 Division of Applied RI, Korea Institute of Radiological and Medical Sciences, Seoul 01812, Korea; osj5353@kirams.re.kr (S.J.O.); krnam@kirams.re.kr (K.R.N.); 4 Department of EMF Research Team, Radio and Broadcasting Technology Laboratory, ETRI, Daejon 34129, Korea; choihd@etri.re.kr

**Keywords:** Alzheimer’s disease, Alzheimer’s disease mice, PLX3397, Aβ pathology, synaptic change, dopamine D2 receptor, colony-stimulating factor 1 receptor inhibitor

## Abstract

Alzheimer’s disease (AD) is a progressive neurodegenerative disease. In this study, to investigate the effect of microglial elimination on AD progression, we administered PLX3397, a selective colony-stimulating factor 1 receptor inhibitor, to the mouse model of AD (5xFAD mice). Amyloid-beta (Aβ) deposition and amyloid precursor protein (APP), carboxyl-terminal fragment β, ionized calcium-binding adaptor molecule 1, synaptophysin, and postsynaptic density (PSD)-95 levels were evaluated in the cortex and hippocampus. In addition, the receptor density changes in dopamine D2 receptor (D2R) and metabotropic glutamate receptor 5 were evaluated using positron emission tomography (PET). D2R, tyrosine hydroxylase (TH), and dopamine transporter (DAT) levels were analyzed in the brains of Tg (5xFAD) mice using immunohistochemistry. PLX3397 administration significantly decreased Aβ deposition following microglial depletion in the cortex and hippocampus of Tg mice. In the neuro-PET studies, the binding values for D2R in the Tg mice were lower than those in the wild type mice; however, after PLX3397 treatment, the binding dramatically increased. PLX3397 administration also reversed the changes in synaptophysin and PSD-95 expression in the brain. Furthermore, the D2R and TH expression in the brains of Tg mice was significantly lower than that in the wild type; however, after PLX3397 administration, the D2R and TH levels were significantly higher than those in untreated Tg mice. Thus, our findings show that administering PLX3397 to aged 5xFAD mice could prevent amyloid pathology, concomitant with the rescue of dopaminergic signaling, suggesting that targeting microglia may serve as a useful therapeutic option for neurodegenerative diseases, including AD.

## 1. Introduction

Aging of the global population has led to a rapid increase in the incidence of Alzheimer’s disease (AD), and there is a growing need for an effective therapeutic drug for AD in order to improve the quality of life of AD patients during old age. AD is a multifactorial neurodegenerative disease that is characterized by progressive cognitive deterioration along with neuropsychiatric symptoms and behavioral changes. There is still a lack of clinical diagnostic parameters for living patients; however, postmortem neuropathologic evaluation has shown that the histopathology of AD is characterized by brain atrophy, amyloid plaques, neurofibrillary tangles, neuron and synapse loss, and dystrophic neurites [1]. Amyloid-beta (Aβ) plaques constitute one of the neuropathological hallmarks of AD and are thought to lead to the neuronal death and synaptic dysfunction that underlie memory impairment [2]. On the basis of the amyloidogenic hypothesis of AD, much research has been conducted on novel pharmacotherapies targeting the Aβ peptide cascade [3]. However, this approach has been unsuccessful in clinical trials, and current research is targeting alternative mechanisms [4].

An increase in the number of reactive microglia is also a key feature of AD [5]. Histopathological studies have shown that activated microglial cells surround amyloid plaques [6,7,8]. Microglia are widely thought to be the key mediators of Aβ clearance in AD [9,10]. However, overwhelming microglial activation exacerbates AD pathology via causing synaptic loss and inflammatory factor secretion [5]. Therefore, controlling microglial reaction in AD progression could serve as a potential therapeutic target for AD.

A few studies reported that depleting microglia with a colony-stimulating factor 1 receptor (CSF1R) inhibitor during the disease process prevented amyloid plaque accumulation in the brains of AD animal models at an early stage of the disease [11,12]. PLX3397 is an orally bioavailable selective CSF1R/c-kit inhibitor that crosses the blood–brain barrier [13]. PLX3397 is already in a Phase 3 clinical study for pigmented villonodular synovitis [14]; however, its therapeutic effects on the late stage of AD have not yet been investigated. Therefore, in this study, we examined the impact of PLX3397 treatment during the late stage of the disease, which involves severe Aβ pathology, by using the 5xFAD mouse model, an AD mouse model.

## 2. Results

### 2.1. PLX3397 Alleviated Aβ Pathology in the Cortex and Hippocampus of Aged Tg Mice

**Figure 1:**
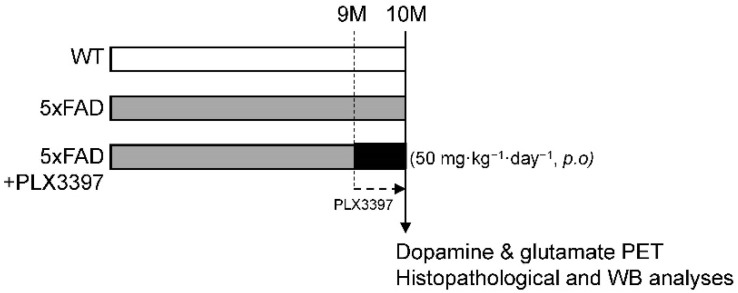
Schematic diagram illustrating the experimental groups and protocol. When the 5xFAD mice were 9 months old, they were administered PLX3397 (50 mg·kg^−1^, *p.o.*) or vehicle for 30 days. Then, each group underwent positron emission tomography (PET) imaging targeting the dopamine D2 receptor (D2R) and metabotropic glutamate receptor 5 (mGluR5), and histopathological and molecular analyses were also performed.

**Figure 2:**
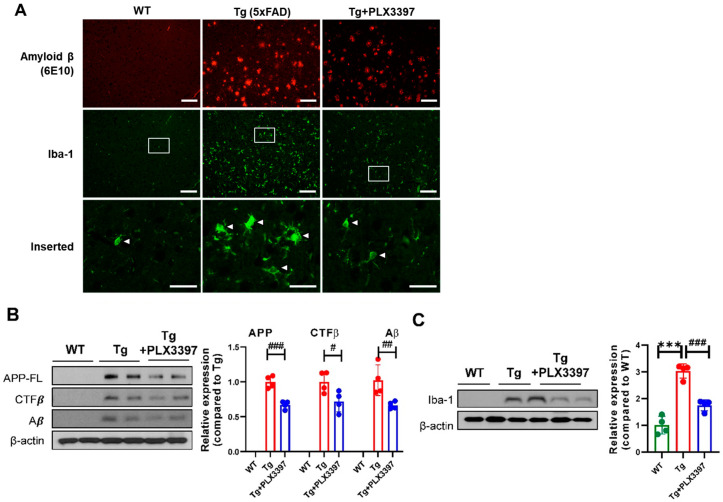
The colony-stimulating factor 1 receptor (CSF1R) inhibitor PLX3397 decreased Aβ levels and amyloid precursor protein (APP) processing in the cortex of 5xFAD mice. (**A**) Representative immunofluorescent images of Amyloid β deposits (6E10 in red) and microglia (Iba-1 in green) in the cortex of 5xFAD mice treated with PLX3397. Arrowhead indicates microglia. Scale bar = 100 μm, inserted image scale bar = 25 μm. (**B**) Representative images of western blots and quantification of expression levels of full-length amyloid-precursor protein (APP-FL), CTFβ, and Aβ in the cortex of WT, Tg, and Tg + PLX3397 mice. (**C**) Representative images of western blots and quantification of Iba-1 expression in the cortex lysate for the indicated groups. Statistical significance has been defined as *** *p* < 0.001 WT vs. Tg; ^#^ *p* < 0.05, ^##^ *p* < 0.01, ^###^ *p* < 0.001 Tg vs. Tg + PLX3397 group. Error bars indicate SD (*n* = 4/group).

To investigate the effect of PLX3397 on a mouse model of AD, 5xFAD mice at the age of 9 months were treated with PLX3397 by daily oral gavage to the mice at 50 mg·kg^−1^ for 30 days (Figure 1). Treatment of the Tg mice with PLX3397 (50 mg·kg^−1^·day^−1^) for 1 month effectively suppressed the microglia in the cortex but did not eliminate them completely (Figure 2A). Oral PLX3397 administration led to a decrease in Aβ deposition in the cortex of the mice. In western blotting analyses, quantification of full-length amyloid precursor protein (APP-FL), carboxyl-terminal fragment β (CTFβ), and Aβ expression revealed 33% (APP-FL; Tg + PLX3397 = 0.669 ± 0.056, *p* = 0.0007), 28% (CTFβ; Tg + PLX3397 = 0.719 ± 0.148, *p* = 0.038), and 33% (Aβ; Tg + PLX3397 = 0.666 ± 0.053, *p* = 0.009) reduction, respectively, in protein levels on using PLX3397 treatment (Figure 2B). Furthermore, the treated mice showed 42% (Iba-1; Tg + PLX3397 = 1.748 ± 0.206, *p* = 0.0003) lower Iba-1 expression in the cortex than the untreated 5xFAD mice (Iba-1; Tg = 3.028 ± 0.269; Figure 2C).

**Figure 3:**
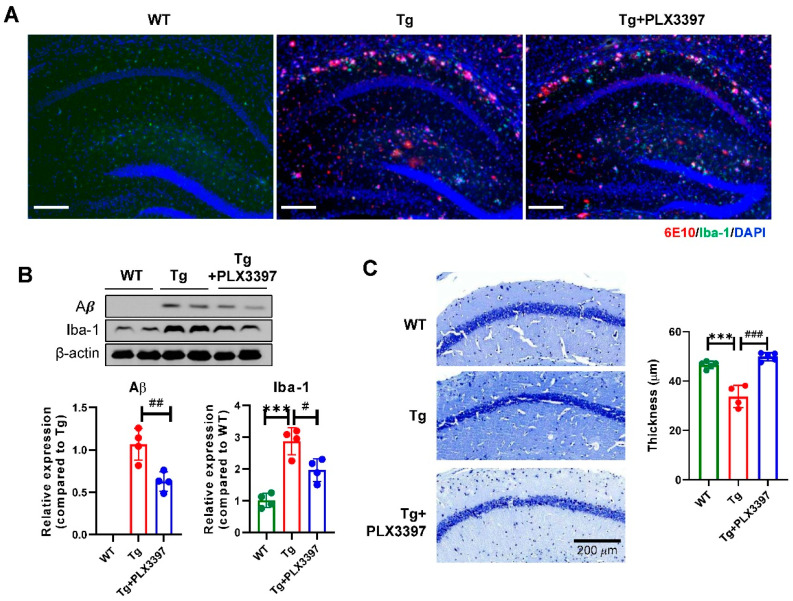
PLX3397 decreased Aβ levels and protected the neuronal cell layer in the hippocampus of 5xFAD mice. (**A**) Representative immunofluorescence image showing staining for Aβ (6E10 in red) and microglia (Iba-1 in green) in the hippocampus. Scale bar = 200 μm. (**B**) Immunoblotting for Aβ and Iba-1 using the hippocampus lysate from each group and Aβ and Iba-1 quantification (*n* = 4/group). (**C**) Representative image of the hippocampal region of 5xFAD mice treated with PLX3397 and quantification of thickness in the CA1 region (*n* = 4 for TG group, *n* = 5 for WT and Tg + PLX3397 group). Scale bar = 200 μm. Statistical significance has been defined as *** *p* < 0.001 WT vs. Tg; ^#^ *p* < 0.05, ^##^ *p* < 0.01, ^###^ *p* < 0.001 Tg vs. Tg + PLX3397 group. Error bars indicate SD.

Next, we analyzed the effect of PLX3397 on the hippocampus of aged Tg mice. In the hippocampus, oral administration of PLX3397 led to lower Aβ load (Aβ; Tg + PLX3397 = 0.624 ± 0.112, *p* = 0.007) and lesser microglial activation (Iba-1; Tg + PLX3397 = 1.959 ± 0.356, *p* = 0.012) compared to those noted for the vehicle-treated Tg group (Iba-1; Tg = 2.868 ± 0.423; Figure 3A,B). 

Because Aβ deposition was significant in the CA1 region, we determined cell layer thickness in CA1 by performing cresyl violet staining. The thickness in the CA1 region in Tg mice (Tg = 33.75 ± 4.49, *p* < 0.0001, WT vs. Tg) was significantly lower than that in wild type (WT) mice (WT = 46.56 ± 1.48) and the cell layer of the CA1 region was protected by PLX3397 administration (Tg + PLX3397 = 49.86 ± 1.64, *p* < 0.0001, Tg vs. Tg + PLX3397; Figure 3C).

### 2.2. PLX3397 Increased Synapse-Related Protein Levels in 5xFAD Mice

**Figure 4:**
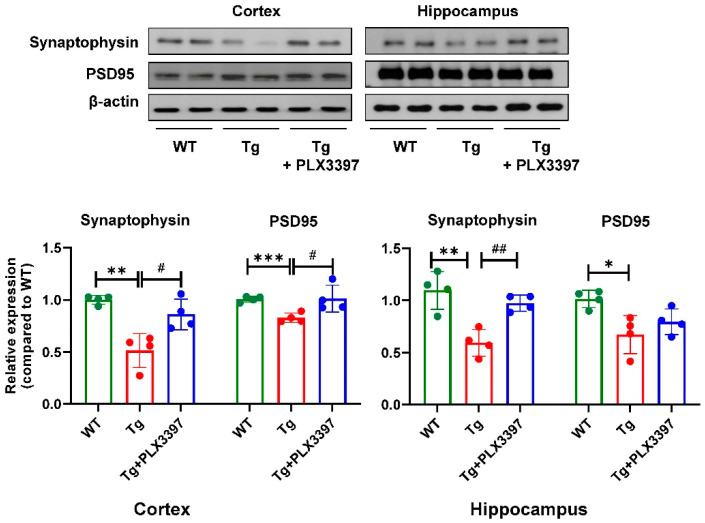
Protective effect of PLX3397 treatment on the levels of synapse plasticity-related proteins in 5xFAD mice. Lysate samples of the vehicle or PLX3397-treated cortex or hippocampus were immunoblotted for synaptophysin, or postsynaptic density (PSD)-95 using specific antibodies. β-actin was used as the loading control. Statistical significance has been defined as * *p* < 0.05, ** *p* < 0.01, *** *p* < 0.001 WT vs. Tg; ^#^ *p* < 0.05, ^##^ *p* < 0.01 Tg vs. Tg + PLX3397 group. Error bars indicate SD (*n* = 4/group).

To determine whether alleviation of Aβ pathology in Tg mice treated with PLX3397 affected synaptic impairment, we analyzed presynaptic and postsynaptic protein levels in the hippocampus and cortex. The Tg mice had significantly lower synaptophysin (Tg = 0.513 ± 0.163, *p* = 0.0012, WT vs. Tg) and postsynaptic density (PSD)-95 (Tg = 33.75 ± 4.49, *p* < 0.0001, WT vs. Tg) levels than those of WT mice in the cortex region. PLX3397 treatment led to significant restoration of synaptophysin levels (Tg + PLX3397 = 0.861 ± 0.147, *p* = 0.0105, Tg vs. Tg + PLX3397) and PSD-95 (Tg + PLX3397 = 1.012 ± 0.129, *p* = 0.0245, Tg vs. Tg + PLX3397) in the cortex. In the hippocampus, the expression levels of synaptophysin (Tg = 0.591 ± 0.128, *p* = 0.0014, WT vs. Tg) and PSD-95 (Tg = 0.671 ± 0.182, *p* = 0.015, WT vs. Tg) was significantly decreased in Tg mice compared with the WT group. Following PLX3397 administration, the levels of synaptophysin (Tg + PLX3397 = 0.973 ± 0.076, *p* = 0.0081, Tg vs. Tg + PLX3397) in the hippocampus was recovered to near-WT levels (Figure 4).

### 2.3. PLX3397 Protected the dopamine (DA) System in Aged 5xFAD Mice

**Figure 5:**
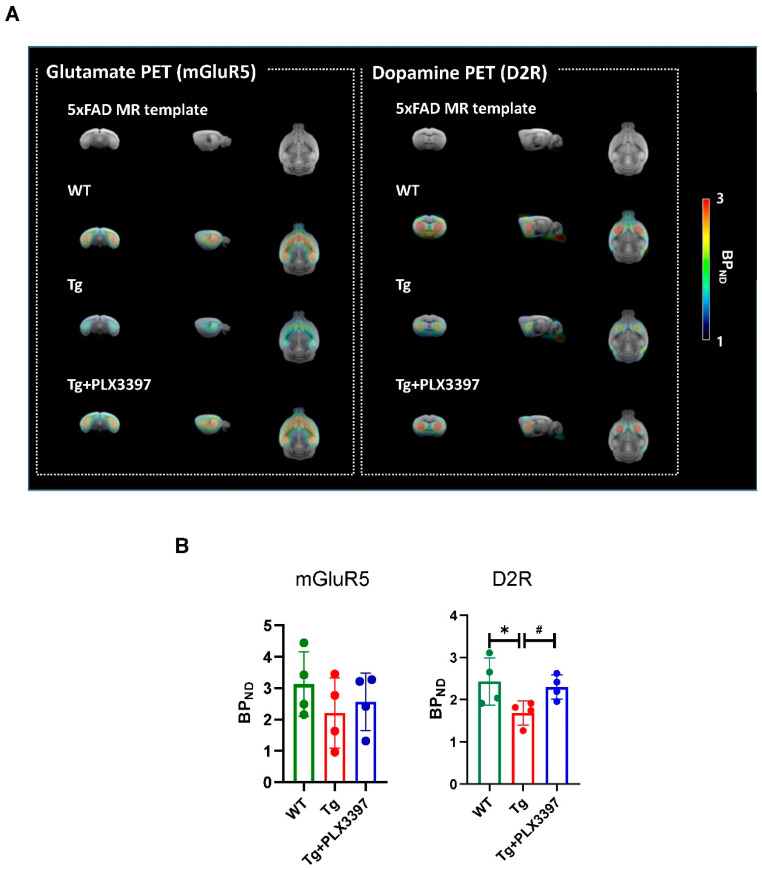
Neuro-PET imaging for evaluating dopamine D2 receptor (D2R) and metabotropic glutamate receptor 5 (mGluR5) expression following PLX3397 treatment. (**A**) Representative nondisplaceable binding potential (BPND) parametric images for WT and Tg mice. Regional brain BPND levels in Tg mice after PLX3397 administration are shown. (**B**) Quantification of BPND for mGluR5 and D2R in the WT, Tg, and Tg + PLX3397 groups. * *p* < 0.05 WT vs. Tg; ^#^ *p* < 0.05 Tg vs. Tg + PLX3397 group. Error bars indicate SD (*n* = 4/group).

To investigate the effect of PLX3397 on synaptic plasticity, we performed neuro-positron emission tomography (PET) imaging for the neurotransmitter receptors dopamine D2 receptor (D2R) and metabotropic glutamate receptor 5 (mGluR5). In DA PET, the radiotracers were predominantly distributed in the striatum whereas in glutamate PET, high radioactivity was detected in the striatum and hippocampus. The nondisplaceable binding potential (BPND) values for D2R and mGluR5 in the Tg group were 31% (Tg = 1.683 ± 0.287, *p* = 0.0142 vs. WT = 2.428 ± 0.558) and 30% (Tg = 2.201 ± 1.119, *p* = 0.3268 vs. WT = 3.125 ± 1.027) lower, respectively, than those for the WT group. However, after PLX3397 administration, the binding values for the Tg + PLX3397 group were 37% (Tg + PLX3397 = 2.296 ± 0.288, *p* = 0.0241, Tg vs. Tg + PLX3397) and 16% (Tg + PLX3397 = 2.553 ± 0.914, *p* = 0.8445, Tg vs. Tg + PLX3397) higher, respectively, than those for the Tg group, although there were no significant differences in the BPND values for mGluR5 after PLX3397 treatment (Figure 5).

**Figure 6:**
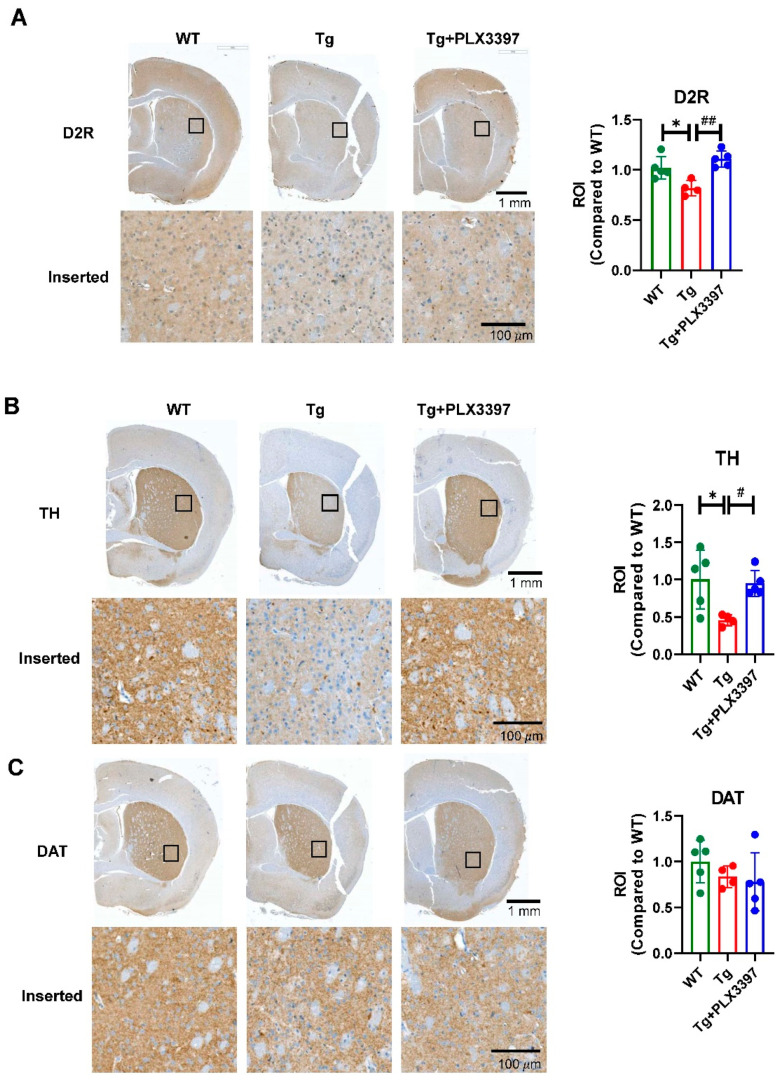
Effects of PLX3397 on the dopaminergic pathway. Representative immunohistochemical images for WT, Tg, and Tg + PLX3397 mice showing staining for dopamine D2 receptor (D2R) (**A**), tyrosine hydroxylase (TH) (**B**), and dopamine transporter (DAT) (**C**) and the quantification of D2R, TH, and DAT levels in the striatum region of the mouse brain (*n* = 4 for TG group, *n* = 5 for WT and Tg + PLX3397 group). * *p* < 0.05 WT vs. Tg; ^#^ *p* < 0.05, ^##^ *p* < 0.01 Tg vs. Tg + PLX3397 group. Error bars indicate SD.

To confirm the alteration of the DA system in Tg mice by PLX3397 treatment, we conducted immunohistochemistry analysis for D2R in the striatum of the mice from the WT, Tg, and Tg + PLX3397 groups. D2R expression in the Tg mice (Tg = 0.818 ± 0.0757, *p* = 0.0187, WT vs. Tg) was lower than that in WT mice, and PLX3397 treatment restored these reduced levels to approximately those observed in case of the WT mice (Tg + PLX3397 = 1.108 ± 0.081, *p* = 0.0017, Tg vs. Tg + PLX3397; Figure 6A), which is consistent with the PET results. Furthermore, we analyzed the tyrosine hydroxylase (TH) and dopamine transporter (DAT) expression levels in the striatum (Figure 6B,C). The expression patterns of DA neurons immunostained with TH were similar to those of DA neurons immunostained with D2R. Tg mice showed significant decreases in TH (Tg = 0.457 ± 0.073, *p* = 0.0254, WT vs. Tg), and PLX3397 prevented this reduction (Tg + PLX3397 = 0.948 ± 0.173, *p* = 0.0423, Tg vs. Tg + PLX3397). However, DAT levels did not significantly differ among the groups (Tg = 0.835 ± 0.116, *p* = 0.9367 vs. Tg + PLX3397 = 0.778 ± 0.316).

## 3. Discussion

In this study, we investigated the impact of PLX3397, a promising CSF1R inhibitor that suppresses microglia, as a new therapeutic option for late AD. On administering PLX3397, activated microglia in the cortex and hippocampus of aged 5xFAD mice were markedly suppressed. Furthermore, PLX3397 effectively suppressed the beta amyloid cascade, including the expression levels of APP, CTFβ, and Aβ peptides, compared to those observed in case of the vehicle-treated group. It is well known that microglia activation is involved in Aβ clearance via phagocytosis during initial AD pathology, however, with the progression of the disease, activated microglia elicit detrimental effects by the overexpression of pro-inflammatory cytokines and decline in Aβ clearance [15,16]. In this study, we have shown that suppressing reactive microglia by PLX3397 treatment exhibited reduced Aβ lesions at the late AD stage. It is thought that suppression of microglia does not directly reduce amyloid accumulation, but it could prevent increases in the accumulation of amyloid by neuroinflammation.

In contrast to some previous studies that investigated the effect of PLX3397 during the early stage of Aβ formation in young 5xFAD mice [11,12], we administered PLX3397 to aged 5xFAD mice with severe AD lesions to determine its therapeutic effects at the late stage of AD. Sosna et al. (2018) [11] showed that 3-month administration of PLX3397 to 2-month-old 5xFAD mice prevented accumulation of intraneuronal Aβ and formation of neuritic plaques. Spangenberg et al. (2016) [12] reported that PLX3397 administration to 1.5-month-old 5xFAD mice reduced memory impairment but did not have any beneficial effects on hippocampal Aβ deposition and Aβ-induced cell death. Moreover, other study reported that 5.5-month administration of PLX5562, a CSF1R inhibitor, to 5xFAD mice at 1.5 months of age prevented the amyloid plaque formation but had no beneficial effect on hippocampal dependent memory [17]. Previous studies have investigated the effect of microglial suppression on the onset and development of AD but our results have suggested the potential of PLX3397 as a therapeutic agent for late-stage or advanced AD.

Synaptic plasticity has been known to play an important role in learning and memory, and dysfunction of synaptic plasticity was demonstrated in AD mice, which were correlated with the Aβ accumulation [18]. Previous studies have reported that PSD-95 levels were reduced in cultured APP mutant neurons compared to wild-type neurons [19]. The expression levels of synaptophysin and PSD-95 were decreased in the mouse model of AD, which were recovered by physical exercise [20]. In the present study, synaptophysin and PSD-95 levels in the brains of vehicle-treated 5xFAD mice were significantly lower than those in age-matched WT mice. PLX3397 significantly increased the levels of synaptophysin proteins both in the cortex and hippocampus. In addition, the level of PSD-95 was reversed after PSD3397 administration in the cortex of 5xFAD mice (Figure 4). To further investigate the effect of PLX3397 on synaptic plasticity, we analyzed the neurotransmitter receptors D2R and mGluR5 by PET imaging involving receptor-specific tracers. Glutamate is the most abundant excitatory neurotransmitter in the central nervous system and regulates learning and memory [21]. mGluR5 dysregulation due to Aβ-synaptic toxicity is related with the early symptoms of cognitive dysfunction in AD [22,23]. The DA system, especially D2R, is a key player in Aβ pathophysiology and neuronal plasticity [24,25]. We previously reported the age dependency of mGluR5 expression in 5xFAD mice at 3, 5, 7, and 9 months of age; we found that 9-month-old 5xFAD mice showed significant decrease in the mGluR5-binding values in the brain, which was consistent with their memory impairment [26]. In the current study, PET analysis confirmed that the expression of mGluR5 tended to decrease in the hippocampus and striatum (Figure 5); PLX3397 treatment showed moderate effect with respect to the protection from the suppression of mGluR5 expression in the hippocampus. Similar to the findings for the mGluR5 status, the brain uptake and binding values of D2R dramatically decreased in the striatum of aged 5xFAD mice. However, compared to the vehicle-treated group, in the PLX3397-treated 5xFAD mice, both uptake and binding of D2R was significantly rescued (Figure 5). Therefore, PLX3397 could have beneficial effects on neuronal plasticity and may have more effects on D2R than on mGluR5. However, further studies are warranted to verify the neurophysiological changes in animal models of AD by long term potentiation measurements and the associated behavioral functions following PLX3397 administration.

DA neurons are considered extremely important in Parkinson’s disease [27], but they have not been of great interest in AD. After neurotransmitter abnormalities were highlighted in AD, the DA system has been intensively studied as a key neurotransmitter system involved with emotion and cognition [28]. Several lines of investigation have shown that dopamine increases cortical excitability by acting through D2-like receptors; these observations support the idea that disruption of the DA system is associated with AD pathophysiology. Currently, dysfunction of DA transmission has been hypothesized as a new player in AD pathophysiology [29]. Consistent with a previous study showing DA neuron loss in the midbrain of 5xFAD mice [25], our study showed that the D2R levels in the striatum of 5xFAD mice were significantly lower than those in the striatum of WT mice. Our finding supported the role of D2R in AD pathology. PET analysis showed that PLX3397 administration rescued this D2R loss in the brains of aged 5xFAD mice. Using IHC, we confirmed this protective effect of PLX3397 on D2R that was noted during PET imaging; loss of both D2R and TH was noted in brain sections of aged 5xFAD mice, which was rescued by PLX3397 treatment (Figure 6). 

Our study has some limitations in that the number of mice in the experimental groups was small because this was a preliminary experiment; furthermore, the effect of PLX3397 on the behavior of the mice is not entirely clear. We performed passive avoidance and object recognition tests after PLX3397 treatment and the results showed a tendency for improved memory function in 5xFAD mice in both behavioral tests; however, these data were not statistically significant owing to the small sample number. Therefore, additional experiments using increased sample sizes are necessary to interpret behavioral changes in response to PLX3397 administration in 5xFAD mice. Although the deposition of amyloid plaques and tau phosphorylation are known to be neuropathological hallmarks of AD, the present study focused on changes in amyloid beta and neuroinflammation expression after PLX3397 treatment owing to lack of tau lesions in 5xFAD mice. Further studies are required to verify the effect of PLX3397 administration on tau pathology as well. Nonetheless, promising effects of PLX3397 were noted in our study, indicating a newly discovered role of PLX3397 that may protect neurotransmission in AD and may apply to other diseases involving DA dysfunction, such as Parkinson’s disease. However, further studies are required to clarify the mechanism underlying the effects of PLX3397 on AD, especially on the DA system of the animals. In addition, subsequent behavioral studies are also needed to elucidate relationship between PLX3397-induced behavioral changes and molecular mechanisms associated with D2R and TH. 

In conclusion, the current study shows that microglial depletion by the CSF1R inhibitor PLX3397 prevented amyloid plaque formation and rescued the expression of synaptic plasticity-related genes in the late stage of AD in 5xFAD mice. The protective effect of PLX3397 on Aβ pathology was consistent with the effects on DA signaling. Therefore, we suggest that the ameliorative effect of PLX3397 on AD progression may be related to the decreased Aβ signaling and recovered levels of synaptic plasticity-related signals, concomitant with the protective effects on DA signaling. Our results may help support the clinical use of the CSF1R inhibitor PLX3397 in AD therapy.

## 4. Materials and Methods 

### 4.1. Animals and Drug Administration

We used transgenic female 5xFAD mice that had five mutant human genes associated with AD: Three APP genes, that is, APPsw, APPfl, and APPlon, and two presenilin1 genes, that is, PS1 and PSEN1 (mutations: PSEN1 M146L and PSEN1 L286V). The method used for generating the 5xFAD mice has been described previously [30]. 5XFAD lines with the B6/SJL genetic background were maintained by crossing hemizygous transgenic mice with B6/SJL F1 breeders. Heterozygous 5xFAD transgenic animals and WT controls were obtained after the breeding of progenitors purchased from the Jackson Laboratory (Jackson Laboratory, Bar Harbor, ME, USA). 

At the age of 9 months, the female 5xFAD mice were assigned to a vehicle control group (Tg: *n* = 4) or PLX3397-treated group (Tg + PLX3397: *n* = 5). Age-matched female B6/SJL mice were assigned to the wild type (WT; *n* = 5). It is known that the pattern of beta amyloid accumulation and neuroinflammation is more severe in female 5xFAD mice than in males [31,32]. Given this sex difference in the severity of AD lesions, we have used only female 5xFAD and age-matched WT mice in our study. PLX3397 (cat no. 206178; MedKoo Biosciences, Morrisville, NC, USA) was dissolved in dimethyl sulfoxide (DMSO) and was then further diluted in an aqueous mixture of 0.5% (hydroxypropyl)methyl cellulose (HPMC; Sigma-Aldrich, St. Louis, MO, USA) and 1% Tween 80 (Sigma-Aldrich). When the Tg mice were 9 months old, 100 µL of the suspended PLX3397 was administered by daily oral gavage to the mice at 50 mg·kg^−1^ for 30 days (Figure 1). The animals were housed in a specific pathogen-free facility and were allowed access to a normal diet and autoclaved water ad libitum. All the mouse procedures in this study were approved by the Institutional Animal Care and Use Committee of the Korea Institute of Radiological and Medical Sciences (IACUC permit number: KIRAMS2018-07, approval date: 27 February 2018).

### 4.2. Immunofluorescence Staining

The tissues were fixed in 4% paraformaldehyde and embedded in paraffin. The deparaffinized and hydrated tissues were incubated in normal goat serum solution (Vector ABC Elite Kit; Vector Laboratories, Burlingame, CA, USA) for 30 min and reacted with the following primary antibodies overnight at 4 °C: 6E10 (cat no. SIG-39320; 1:1000 dilution; Biolegend, San Diego, CA, USA) and ionized calcium-binding adaptor molecule 1 (Iba-1; cat no. 019-19741; 1:200 dilution; Wako, Neuss, Germany). Subsequently, the tissues were washed with phosphate-buffered saline (PBS) containing 0.1% Triton-X 100 (PBS-Triton-X) and tagged with tetramethylrhodamine isothiocyanate-labeled anti-mouse or anti-rabbit IgG antibody for 30 min at room temperature. The tissues were washed in PBS-Triton-X again, counterstained with 4′,6-diamidino-2-phenylindole (DAPI), and mounted. The stained tissues were observed under a fluorescence microscope.

### 4.3. Immunohistochemistry

The tissues were deparaffinized, hydrated, and washed with PBS-Triton-X. For the block nonspecific staining, the tissues were incubated in normal goat serum (Vector ABC Elite Kit) at room temperature for 30 min. The washed tissues were then incubated with the following primary antibodies overnight at 4 °C: Anti-rabbit D2R (cat no. AB5084P; 1:500 dilution; Merck, Darmstadt, Germany), anti-rabbit TH (cat no. NB300-109; 1:1000 dilution; Novus, Centennial, CO, USA), or anti-rabbit DAT (cat no. D6944; 1:000 dilution; Sigma-Aldrich) antibodies. Subsequently, they were washed and incubated with biotinylated secondary antibody for 30 min at room temperature. After incubation, the tissues were incubated with an avidin–biotin–peroxidase complex (Vector Laboratories) for 30 min and incubated with diaminobenzidine (DAB, Vector Laboratories). Images were obtained at 20× magnification by using whole slide digital scanning with a Digital Pathology Scanner (Aperio VERSA; Leica Biosystems, Buffalo Grove, IL, USA), and the positive cells were counted using Aperio Image Analysis.

### 4.4. Cresyl Violet Staining

The 5 m tissue sections were deparaffinized, hydrated using a series of graded ethanol solutions, and washed in tap water for 5 min. The tissues were incubated in 0.5% cresyl violet solution for 20 min and differentiated in 0.25% acetic alcohol. Subsequently, they were dehydrated in graded ethanol and two changes of xylene for 5 min. The tissues slides were cover-slipped using mounting medium.

### 4.5. Western Blotting

The tissues were homogenized in radioimmunoprecipitation (RIPA) buffer containing a protease inhibitor and phosphatase inhibitor. The protein concentration was determined using Bradford reagent (Bio-Rad, Hercules, CA, USA). Equal amounts of the total protein (10–30 μg) were electrophoresed on 8–12.5% sodium dodecyl sulfate (SDS)–polyacrylamide gels and transferred to nitrocellulose membranes. The membranes were incubated in 3% bovine serum albumin (BSA) solution for nonspecific blocking, followed by overnight incubation at 4 °C with the primary antibody. The following primary antibodies and dilutions were used: Anti-6E10 (cat no. SIG-39320; 1:1000 dilution; Biolegend), anti-Iba-1 (cat no. 106-20001; 1:1000 dilution; Wako), anti-synaptophysin (cat no. MAB-5258; 1:1000 dilution; Merck), anti-PSD-95 (cat no. GTX133091; 1:1000 dilution; GeneTex, Irvine, CA, USA), and anti-β-actin (cat no. A1978; 1:2000 dilution; Sigma-Aldrich) antibodies. Subsequently, the membranes were washed in PBS containing 0.1% Tween 20 and incubated with a 1:3000 dilution of horseradish peroxidase (HRP)-conjugated secondary antibodies (Santa Cruz Biotechnology, Dallas, TX, USA) for 1 h at room temperature. The membranes were then washed and developed using an enhanced chemiluminescence (ECL) kit (Perkin Elmer, Waltham, MA, USA).

### 4.6. PET Scans

We used two kinds of radiotracers, that is, (S)-N-[(1 allyl-2-pyrrolidinyl)methyl]-5-(3[^18^F]fluoropropyl)-2,3-dimethoxybenzamide ([^18^F]fallypride) and [^18^]F-3-fluoro-5-[(pyridin-3-yl)ethynyl]benzonitrile ([^18^F]FPEB), which are specific radiotracers for D2R and mGluR5, respectively. These radiotracers were synthesized by F-18 radiolabeling of tosyl or nitro precursors, according to a previously described procedure [33,34]. The radiochemical purity at the end of synthesis was over 95%. 

The PET experiments were conducted using an animal-dedicated PET scanner (nanoScan; Mediso Medical Imaging Systems, Budapest, Hungary). The scanner has a peak absolute system sensitivity of >9% in the 250–750 keV energy window, an axial field of view of 28 cm, a transaxial field of view of 35–120 mm, and a transaxial resolution of 0.7 mm at 1 cm off-center. 

All the mice were anesthetized with 2.5% isoflurane in oxygen, and 8.5 ± 0.7 MBq [^18^F]fallypride and [^18^F]FPEB were administered via the tail vein. PET scanning was performed for 90 min in the list mode. Subsequently, PET data were reconstructed in user-defined time frames (10 s × 6 frames, 30 s × 8 frames, 180 s × 5 frames, and 600 s × 7 frames) with voxel dimensions of 0.86 × 0.86 × 0.80 mm^3^ with a three-dimensional ordered-subset expectation maximization algorithm (four iterations and six subsets). For attenuation correction, micro-CT imaging was conducted immediately after PET scanning, using a 50 kVp X-ray voltage with 0.16 mAs.

### 4.7. PET Image Analysis

After mean PET images (50–90 min) were obtained from the dynamic PET images, each mean PET image was spatially normalized to our own tracer-specific brain magnetic resonance (MR) template [26], using the PMOD software (version 3.8, PMOD Technologies Ltd. Zürich, Switzerland). Subsequently, individual normalization parameters were applied to the dynamic PET images and brain masking was applied. The standardized uptake value (SUV) images were obtained by normalizing tissue radioactivity concentration by injected dose and body weight.

Two volumes of interests (VOIs) of the striatum and cerebellum were defined on the MR template. The cerebellum was used as a reference region because it has been shown to have very low D2R [35,36] and mGluR5 [37,38] levels. Next, the decay-corrected regional time–activity curves (TACs) of two regions were obtained from the VOIs. 

To estimate the receptor density in vivo, we derived the BPND by noninvasive Logan graphical analysis [39]. The clearance rate (k2′) of the reference tissue was estimated with the simplified reference tissue model (SRTM) and then applied to each BPND calculation. Voxel-based parametric mapping was also utilized with Logan’s method. For each group, the individual parametric map was averaged. Then, the mean PET image was coregistered to study the specific MR template in order to obtain a PET–MR image.

### 4.8. Statistical Analysis 

The data have been expressed in terms of mean ± standard deviation (SD). Statistical significance was determined using one-way analysis of variance (ANOVA) followed by the Tukey multiple comparison test by using the GraphPad Prism program (version 8.0, GraphPad Software, Inc. San Diego, CA, USA). A *p* value of <0.05 was considered significant. Differences between the groups in PET imaging (*n* = 4) were tested using the Mann–Whitney nonparametric test, with *p* < 0.05 being considered to indicate significant difference.

## References

[1] A DeTure M., Dickson D.W. (2019). The Neuropathological Diagnosis of Alzheimer’s Disease. Mol. Neurodegener..

[2] Hsia A.Y., Masliah E., McConlogue L., Yu G.Q., Tatsuno G., Hu K., Kholodenko D., Malenka R.C., Nicoll R.A., Mucke L. (1999). Plaque-independent disruption of neural circuits in Alzheimer’s disease mouse models. Proc. Natl. Acad. Sci. USA.

[3] Folch J., Petrov D., Ettcheto M., Abad S., Sanchez-Lopez E., Garcia M.L., Olloquequi J., Beas-Zarate C., Auladell C., Camins A. (2016). Current Research Therapeutic Strategies for Alzheimer’s Disease Treatment. Neural Plast..

[4] Khan A., Corbett A., Ballard C. (2017). Emerging treatments for Alzheimer’s disease for non-amyloid and non-tau targets. Expert Rev. Neurother..

[5] Hansen D.V., Hanson J.E., Sheng M. (2018). Microglia in Alzheimer’s disease. J. Cell Biol..

[6] Dani M., Wood M., Mizoguchi R., Fan Z., Walker Z., Morgan R., Hinz R., Biju M., Kuruvilla T., Brooks D.J. (2018). Microglial activation correlates in vivo with both tau and amyloid in Alzheimer’s disease. Brain J. Neurol..

[7] Majerova P., Zilkova M., Kazmerova Z., Kovac A., Paholikova K., Kovacech B., Zilka N., Novak M. (2014). Microglia display modest phagocytic capacity for extracellular tau oligomers. J. Neuroinflammation.

[8] Perlmutter L.S., Barron E., Chui H.C. (1990). Morphologic association between microglia and senile plaque amyloid in Alzheimer’s disease. Neurosci. Lett..

[9] Sarlus H., Heneka M.T. (2017). Microglia in Alzheimer’s disease. J. Clin. Invest..

[10] Rivera-Escalera F., Pinney J.J., Owlett L., Ahmed H., Thakar J., Olschowka J.A., Elliott M.R., O’Banion M.K. (2019). IL-1beta-driven amyloid plaque clearance is associated with an expansion of transcriptionally reprogrammed microglia. J. Neuroinflammation.

[11] Sosna J., Philipp S., Albay R., Reyes-Ruiz J.M., Baglietto-Vargas D., LaFerla F.M., Glabe C.G. (2018). Early long-term administration of the CSF1R inhibitor PLX3397 ablates microglia and reduces accumulation of intraneuronal amyloid, neuritic plaque deposition and pre-fibrillar oligomers in 5XFAD mouse model of Alzheimer’s disease. Mol. Neurodegener..

[12] Spangenberg E.E., Lee R.J., Najafi A.R., Rice R.A., Elmore M.R., Blurton-Jones M., West B.L., Green K.N. (2016). Eliminating microglia in Alzheimer’s mice prevents neuronal loss without modulating amyloid-beta pathology. Brain J. Neurol..

[13] Elmore M.R., Najafi A.R., Koike M.A., Dagher N.N., Spangenberg E.E., Rice R.A., Kitazawa M., Matusow B., Nguyen H., West B.L. (2014). Colony-stimulating factor 1 receptor signaling is necessary for microglia viability, unmasking a microglia progenitor cell in the adult brain. Neuron.

[14] Cannarile M.A., Weisser M., Jacob W., Jegg A.M., Ries C.H., Ruttinger D. (2017). Colony-stimulating factor 1 receptor (CSF1R) inhibitors in cancer therapy. J. Immunother. Cancer.

[15] Kaur D., Sharma V., Deshmukh R. (2019). Activation of microglia and astrocytes: A roadway to neuroinflammation and Alzheimer’s disease. Inflammopharmacology.

[16] Li J.W., Zong Y., Cao X.P., Tan L., Tan L. (2018). Microglial priming in Alzheimer’s disease. Annu. Transl. Med..

[17] Spangenberg E., Severson P.L., Hohsfield L.A., Crapser J., Zhang J., Burton E.A., Zhang Y., Spevak W., Lin J., Phan N.Y. (2019). Sustained microglial depletion with CSF1R inhibitor impairs parenchymal plaque development in an Alzheimer’s disease model. Nat. Commun..

[18] Oddo S., Caccamo A., Shepherd J.D., Murphy M.P., Golde T.E., Kayed R., Metherate R., Mattson M.P., Akbari Y., LaFerla F.M. (2003). Triple-transgenic model of Alzheimer’s disease with plaques and tangles: Intracellular Abeta and synaptic dysfunction. Neuron.

[19] Almeida C.G., Tampellini D., Takahashi R.H., Greengard P., Lin M.T., Snyder E.M., Gouras G.K. (2005). Beta-amyloid accumulation in APP mutant neurons reduces PSD-95 and GluR1 in synapses. Neurobiol. Dis..

[20] Revilla S., Sunol C., Garcia-Mesa Y., Gimenez-Llort L., Sanfeliu C., Cristofol R. (2014). Physical exercise improves synaptic dysfunction and recovers the loss of survival factors in 3xTg-AD mouse brain. Neuropharmacology.

[21] Collingridge G.L., Singer W. (1990). Excitatory amino acid receptors and synaptic plasticity. Trends Pharmacol. Sci..

[22] Ferreira S.T., Klein W.L. (2011). The Abeta oligomer hypothesis for synapse failure and memory loss in Alzheimer’s disease. Neurobiol. Learn. Mem..

[23] Revett T.J., Baker G.B., Jhamandas J., Kar S. (2013). Glutamate system, amyloid ss peptides and tau protein: Functional interrelationships and relevance to Alzheimer disease pathology. J. Psychiatry Neurosci..

[24] Donthamsetti P., Gallo E.F., Buck D.C., Stahl E.L., Zhu Y., Lane J.R., Bohn L.M., Neve K.A., Kellendonk C., Javitch J.A. (2018). Arrestin recruitment to dopamine D2 receptor mediates locomotion but not incentive motivation. Mol. Psychiatry.

[25] Vorobyov V., Bakharev B., Medvinskaya N., Nesterova I., Samokhin A., Deev A., Tatarnikova O., Ustyugov A., Sengpiel F., Bobkova N. (2019). Loss of Midbrain Dopamine Neurons and Altered Apomorphine EEG Effects in the 5xFAD Mouse Model of Alzheimer’s Disease. J. Alzheimer’s Dis..

[26] Lee M., Lee H.J., Jeong Y.J., Oh S.J., Kang K.J., Han S.J., Nam K.R., Lee Y.J., Lee K.C., Ryu Y.H. (2019). Age dependency of mGluR5 availability in 5xFAD mice measured by PET. Neurobiol. Aging.

[27] Michel P.P., Hirsch E.C., Hunot S. (2016). Understanding Dopaminergic Cell Death Pathways in Parkinson Disease. Neuron.

[28] Nardone R., Höller Y., Thomschewski A., Kunz A.B., Lochner P., Golaszewski S., Trinka E., Brigo F. (2014). Dopamine differently modulates central cholinergic circuits in patients with Alzheimer disease and CADASIL. J. Neural. Transm. (Vienna).

[29] Nam E., Derrick J.S., Lee S., Kang J., Han J., Lee S.J.C., Chung S.W., Lim M.H. (2018). Regulatory Activities of Dopamine and Its Derivatives toward Metal-Free and Metal-Induced Amyloid-beta Aggregation, Oxidative Stress, and Inflammation in Alzheimer’s Disease. ACS Chem. Neurosci..

[30] Son Y., Kim J.S., Jeong Y.J., Jeong Y.K., Kwon J.H., Choi H.D., Pack J.K., Kim N., Lee Y.S., Lee H.J. (2018). Long-term RF exposure on behavior and cerebral glucose metabolism in 5xFAD mice. Neurosci. Lett..

[31] Manji Z., Rojas A., Wang W., Dingledine R., Varvel N.H., Ganesh T. (2019). 5xFAD Mice Display Sex-Dependent Inflammatory Gene Induction During the Prodromal Stage of Alzheimer’s Disease. J. Alzheimer’s Dis..

[32] Sadleir K.R., Popovic J., Vassar R. (2018). ER stress is not elevated in the 5XFAD mouse model of Alzheimer’s disease. J. Biol. Chem..

[33] Moon B.S., Park J.H., Lee H.J., Kim J.S., Kil H.S., Lee B.S., Chi D.Y., Lee B.C., Kim Y.K., Kim S.E. (2010). Highly efficient production of [(18)F]fallypride using small amounts of base concentration. Appl. Radiat. Isot..

[34] Lee M., Lee H.J., Park I.S., Park J.A., Kwon Y.J., Ryu Y.H., Kim C.H., Kang J.H., Hyun I.Y., Lee K.C. (2018). Abeta pathology downregulates brain mGluR5 density in a mouse model of Alzheimer. Neuropharmacology.

[35] Boja J.W., Mitchell W.M., Patel A., Kopajtic T.A., Carroll F.I., Lewin A.H., Abraham P., Kuhar M.J. (1992). High-affinity binding of [125I]RTI-55 to dopamine and serotonin transporters in rat brain. Synapse.

[36] Cline E.J., Scheffel U., Boja J.W., Mitchell W.M., Carroll F.I., Abraham P., Lewin A.H., Kuhar M.J. (1992). In vivo binding of [125I]RTI-55 to dopamine transporters: Pharmacology and regional distribution with autoradiography. Synapse.

[37] Elmenhorst D., Minuzzi L., Aliaga A., Rowley J., Massarweh G., Diksic M., Bauer A., Rosa-Neto P. (2010). In vivo and in vitro validation of reference tissue models for the mGluR(5) ligand [(11)C]ABP688. J. Cereb. Blood Flow Metab..

[38] Shigemoto R., Nomura S., Ohishi H., Sugihara H., Nakanishi S., Mizuno N. (1993). Immunohistochemical localization of a metabotropic glutamate receptor, mGluR5, in the rat brain. Neurosci. Lett..

[39] Logan J., Fowler J.S., Volkow N.D., Wolf A.P., Dewey S.L., Schlyer D.J., MacGregor R.R., Hitzemann R., Bendriem B., Gatley S.J. (1990). Graphical analysis of reversible radioligand binding from time-activity measurements applied to [N-11C-methyl]-(-)-cocaine PET studies in human subjects. J. Cereb. Blood Flow Metab..

