# Expression of Concern: Son et al. Inhibition of Colony-Stimulating Factor 1 Receptor by PLX3397 Prevents Amyloid Beta Pathology and Rescues Dopaminergic Signaling in Aging 5xFAD Mice. *Int. J. Mol. Sci.* 2020, *21*, 5553

**DOI:** 10.3390/ijms27209304

**Published:** 2026-10-20

**Authors:** International Journal of Molecular Sciences Editorial Office

**Affiliations:** MDPI, St. Alban-Anlage 66, 4052 Basel, Switzerland; ijms@mdpi.com

With this notice, the IJMS Editorial Office alerts readers to concerns regarding this article [1]. Following publication, concerns were raised about apparent duplication within Figure 6B. The authors were contacted and asked to provide an explanation and the original data to address these concerns. However, no response was received, and the matter could therefore not be conclusively resolved.

The Editorial Board assessed the available information and concluded that, based on the evidence currently available, the concerns do not appear to substantially affect the overall conclusions of the publication.

This Expression of Concern is issued to inform readers of the unresolved concerns relating to Figure 6B. No further action is planned at this time. However, should additional information become available, further action may be taken in accordance with MDPI’s policies if deemed necessary.
